# Effect of DNA extraction procedure, repeated extraction and ethidium monoazide (EMA)/propidium monoazide (PMA) treatment on overall DNA yield and impact on microbial fingerprints for bacteria, fungi and archaea in a reference soil

**DOI:** 10.1016/j.apsoil.2015.04.005

**Published:** 2015-09

**Authors:** Andreas O. Wagner, Nadine Praeg, Christoph Reitschuler, Paul Illmer

**Affiliations:** Institute of Microbiology, University of Innsbruck, Technikerstr. 25, A-6020 Innsbruck, Austria

**Keywords:** DNA-extraction, Repeated extraction, Ethidium monoazide (EMA), Propidium monoazide (PMA), Diversity

## Abstract

Different DNA extraction protocols were evaluated on a reference soil. A wide difference was found in the total extractable DNA as derived from different extraction protocols. Concerning the DNA yield phenol–chloroform–isomyl alcohol extraction resulted in high DNA yield but also in a remarkable co-extraction of contaminants making PCR from undiluted DNA extracts impossible. By comparison of two different extraction kits, the Macherey&Nagel SoilExtract II kit resulted in the highest DNA yields when buffer SL1 and the enhancer solution were applied. The enhancer solution not only significantly increased the DNA yield but also the amount of co-extracted contaminates, whereas additional disintegration strategies did not. Although a three times repeated DNA extraction increased the total amount of extracted DNA, microbial fingerprints were merely affected. However, with the 5th extraction this changed. A reduction of total DGGE band numbers was observed for archaea and fungi, whereas for bacteria the diversity increased. The application of ethidium monoazide (EMA) or propidium monoazide (PMA) treatment aiming on the selective removal of soil DNA derived from cells lacking cell wall integrity resulted in a significant reduction of total extracted DNA, however, the hypothesized effect on microbial fingerprints failed to appear indicating the need for further investigations.

## Highlights

• We evaluated different soil DNA extraction procedures and disintegration strategies.
• All tests were conducted using a reference soil.
• We tested repeated DNA extractions (up to 10 times).
• A method (EMA; PMA) for the discrimination of cells and free DNA was tested.
• DNA yield is affected by extraction procedure, microbial diversity merely.

## 1 Introduction

For a long time classic-microbiological, cultivation-based methods have been the only way to investigate and describe a microbial community. The establishment of polymerase chain reaction (PCR) – a technique allowing the amplification of minute amounts of DNA very rapidly and to such an extent that the DNA becomes easy to detect – has altered biological science radically, thus molecular biological methods became essential for the culture independent investigation of the microbiota of different habitats and have revolutionized our understanding of microbial community structure and diversity within the environment (Hazen et al., 2013, Taberlet et al., 2012). Different fingerprinting methods such as DGGE, TGGE, SSCP, RFLP, dHPLC etc., but also clone libraries and highly modern sequencing techniques are currently used to investigate and characterize microbial populations in various habitats. However, the fundament of all these molecular methods is based on a successful, complete but also not false-positive extraction of nucleic acids. Numerous different extraction protocols and kits are used for different environmental samples making it difficult to compare results quantitatively and qualitatively (Smith and Osborn, 2009).

Soil as a very complex and heterogeneous microbial habitat makes a reproducible extraction of DNA challenging as soil DNA is in a permanent turn-over and extracellular DNA is cycled (Levy-Booth et al., 2007). Moreover, DNA extraction methods are often biased by various, often undefined parameters and by the fact, that the discrimination between intracellular DNA of viable cells and unprotected DNA (“free DNA”) is hardly possible. Otherwise, RNA based approaches, which would target the active part of a microbial community, thus enabling discrimination between living and dead cells, experience problems with the fast RNA-decay rates after loss of cell viability (Belasco, 1993) and are more expensive and laborious with respect to DNA based analysis. Therefore, besides the requested intracellular DNA, extracellular nucleic acids are extracted as well via extraction procedures and may contribute considerably to extracted amounts of total DNA (Niemeyer and Gessler, 2002, Ceccherini et al., 2009, Ascher et al., 2009). Thus, the actual microbial composition of the sample is likely being misrepresented. Most molecular environmental studies do not deal with this fact so far, although the presence of extracellular DNA is known for many years (Ogram et al., 1994, Levy-Booth et al., 2007, Pietramellara et al., 2009). This is remarkable, as the extraction of DNA is one of the basic steps in molecular ecological approaches and one can imagine that all subsequent molecular analyses e.g., PCR, DGGE, cloning, (NGS)-sequencing etc. might be biased right away from the start.

In the present study the reduction of the portion of extractable DNA not derived from soil microorganisms maintaining cell wall integrity was evaluated by the application of ethidium monoazide (EMA) and propidium monoazide (PMA) treatment, respectively, a method that was shown to be successful with clinical samples (Nocker and Camper, 2006, Nocker and Camper, 2009, Rudi et al., 2005) and promising with environmental samples (Elizaquivel et al., 2014, Lee and Levin, 2007, Shi et al., 2011, van Frankenhuyzen et al., 2013, Wagner et al., 2008). Prior to DNA extraction EMA or PMA is added to the desired extraction matrix and after photolysis the chemicals covalently bind to DNA, by conversion of the azide group into a nitrene radical (Trevors, 2012). Since EMA and PMA are hypothesized to not permeate cytoplasmic membranes (Nocker et al., 2006), by this method unprotected DNA or DNA of microorganisms not maintaining cell wall integrity is removed and the active (alive) part of the microbial community of a certain habitat can be addressed. As far as we know this method has not been used for soil DNA extraction yet.

The aim of this work was to evaluate different DNA extraction methods, protocols, and commercially available kits, respectively, with respect to (i) total DNA yield, (ii) the quality of the obtained DNA, (iii) possible bias of PCR amplification caused by the choice of DNA extraction method/protocol and (iv) the effect of EMA- and PMA-treatment on total DNA yield, reduced co-extraction of unprotected DNA, and its effect on microbial fingerprints. All analyses were performed using a reference soil.

## 2 Material and methods

### 2.1 Reference soil

For this study a reference soil derived from Landwirtschaftliche Untersuchungs- und Forschungsanstalt Speyer, Germany, was used (http://www.lufa-speyer.de/). It is defined as reference soil 2.3 for soil type silty sand (uS) (after German DIN) or sandy loam (after U.S. Department of Agriculture). Basic soil parameters are given in Table 1.

**Table 1:** Basic soil parameters of the used reference soil according to Landwirtschaftliche Untersuchungs- und Forschungsanstalt Speyer, Germany.

Soil parameter	Mean (±SD)
Organic carbon in % C	0.94 (0.10)
Nitrogen in % N	0.08 (0.02)
pH	6.8 (0.2)
Cation exchange capacity (meq 100 g^−1^)	10.9 (1.1)
Soil type according German DIN	Silty sand (uS)
Soil type according USDA	Sandy loam
Water holding capacity (w/w) (g 100 g^−1^)	37.3 (1.8)
Water holding capacity (w/v) (g 1000 ml^−1^)	1282 (30)

### 2.2 DNA extraction procedures

**Fig. 1:**
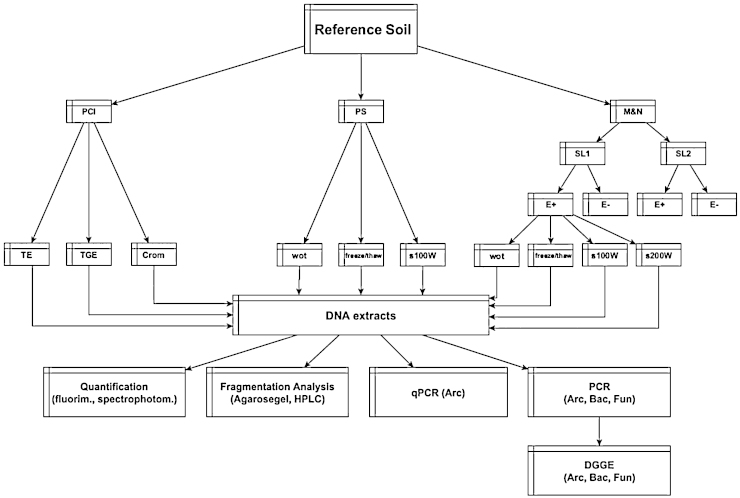
Scheme of applied extraction protocols, buffers, enhancer, and disintegration strategies. PS: PowerSoil kit; M&N: SoilExtract II kit; PCI: phenol–chlorophorm–isoamyl extraction; wot: without treatment; SL1, SL2, E: M&N provided buffers and enhancer solution (E+: with; E−: without enhancer); s100 W: sonic treatment 100 W; s200 W: sonic treatment 200 W; TE: Tris–EDTA buffer; TGE: glucose–Tris–EDTA buffer; Crom: Crombach buffer.

For DNA extractions 250 mg of soil sample was processed at least in triplicate. Two commercially available soil-DNA extraction kits (SoilExtract II, Macherey&Nagel, M&N; PowerSoil DNA extraction Kit MO BIO Lab-Inc., PS) were tested following manufacturers’ protocols. Both kits consist of a beat-beating step for enhanced cell disruption. For M&N the two provided extraction buffers (SL1 and SL2) were tested as well as several concentrations of enhancer solution. Moreover, standard phenol–chloroform extraction was performed. This methodology nowadays is very rarely or not used anymore for microbial diversity studies but was included to also test a method not applying a commercial kit. Phenol–chloroform extraction generally followed the procedure proposed by Maciel et al. (2009) including a lysozyme treatment (2.5 mg ml^−1^) and a beat beating step (beat diameter 1 mm, 10 min beating time). Different extraction buffers were tested (TE buffer: 50 mM Tris–HCl, 50 mM EDTA, pH 8; Crombach buffer (Crom): 33 mM Tris–HCl, 1 mM EDTA, pH 8 (Krsek and Wellington, 1999), TEG buffer: 50 mM glucose, 10 mM EDTA, 25 mM Tris–HCl, pH 8). DNA derived from the different protocols was eluted in 25–100 μl of 5 mM Tris–EDTA buffer, pH 8.5. Additionally, various disintegration strategies were tested (freeze/thaw cycles, sonic treatment 100 W, sonic treatment 200 W). An additional scheme of the extraction procedure in combination with buffers, enhancers and disintegration strategies is provided in Fig. 1.

In order to assess the efficiency of the protocols, repeated DNA extractions were performed for PS and M&N (also in triplicate). For this purpose the protocol was repeated on the same sample but without the beat-beating step and the elution volume was reduced to 25 μl. For PCI extractions a repeated extraction was omitted since a co-extraction of various “contaminants” was obvious after the first tests (PCR inhibition).

Fragmentation of DNA was evaluated via 1% gel electrophoresis (100 V, 25 min). Alternatively, an aliquot of DNA extract was loaded on a dHPLC system as described in Wagner et al. (2009), applying non-denaturing conditions (50 °C). A gradient of 95% buffer A (100 mM triethyl-ammonium-acetate aqueous solution) and 5% buffer B (25% acetonitrile in 100 mM triethyl-ammonium-acetate aqueous solution) to 100% buffer B was run in 20 min. Detection was carried out via a UV–vis detector at 254 nm.

### 2.3 Disintegration strategies

To enhance cell lysis samples were subjected to different disintegration strategies: (i) 3 cycles of freezing (1 h at −80 °C) and thawing (30 min at 37 °C) after the bead-beating step, (ii) sonic treatment using a 100 W ultrasonic bath (Elmasonic, Elma), and (iii) sonic treatment (200 W) with a special sonication device (Bandelin Sonopuls HD 200 equipped with a HW 200). The sonication was repeated three times for 60 s, 40 s, and 20 s, respectively, in the ultrasonic bath (100 W) and for 30 s each for the sonication device (200 W).

### 2.4 EMA and PMA treatment

In order to evaluate the impact of an additional treatment with ethidium monoazide (EMA) or propidium monoazide (PMA) during DNA extraction for a selective removal of unprotected DNA, 100 μM EMA or PMA (Biotium, VWR, Germany) were applied as described previously (Wagner et al., 2008). After EMA or PMA addition the samples were vortexed for 5 s and incubated for 10 min in the dark in order to allow EMA and PMA, respectively, to penetrate the cells with damaged cell walls, followed by a 30 min light incubation (650 W halogen) for the activation of EMA and PMA, respectively, by binding to DNA. In order to avoid the samples getting heated, light activation was done on ice. Samples were mixed very gently in 5 min intervals. Following to EMA/PMA treatment the DNA extraction procedure was conducted according to the respective protocol starting with the beat-beating step.

### 2.5 DNA quantification

The extracted DNA was quantified using two different methods, on the one hand fluorometrically with PicoGreen dsDNA quantification reagent (Invitrogen, Carlsbad, USA, Anthos-Zenyth Multimode Detector) following the procedure of (Juen and Traugott, 2005) and on the other hand spectrophotometrically with NanoDrop 2000c™ (PEQLAB, Germany) (Abs at 260 nm = 1.0 referred to a concentration of 50 ng μl^−1^), where also the quality of the DNA extract was evaluated. Humic substance co-extraction was assessed using the absorbance ratio A260/230, whereas the ratio A260/280 indicated co-extraction of proteins and high amounts of RNA.

### 2.6 PCR-DGGE and qPCR

PCR was performed using primers for archaea (787fGC/1059r), bacteria (338fGC/805r) (Yu et al., 2004), and fungi (FR1fGC/FF390) (Vainio and Hantula, 2000). PCR conditions for archaeal primers can be found in Reitschuler et al. (2013), for bacterial ones in Reitschuler et al. (2014), and for fungi in Vainio and Hantula (2000). For archaeal and bacterial primers an initial denaturation for 300 s at 95 °C followed by 35 cycles of denaturation for 30 s at 95 °C, annealing for 30 s at 57 °C, and elongation for 30 s at 72 °C with a final elongation for 420 s (for archaeal primers) and 600 s (for bacterial primers) at 72 °C was applied. Fungal PCR was conducted under the following conditions: 95 °C for initial denaturation for 300 s, followed by 35 cycles of 30 s at 95 °C, 30 s at 50 °C, and 60 s at 72 °C, and completed by a final elongation step of 300 s at 72 °C. The PCR mix contained 12.5 μl PCR MyTaq 2× PCR MasterMix (VWR, Germany), 250 nM primer, 4% (v/v) of enhancer solution (TaqMaster Enhancer solution 5×, VWR, Germany) and molecular grade PCR water to obtain a final volume of 25 μl. A total of 1.5 μl of template DNA was added to 23.5 μl PCR mix for all tested protocols but not for PCI extraction, where the DNA extract required a 1:25 dilution for its PCR compatibility. Quantity and quality of amplified DNA were assessed via gel electrophoresis prior to further processing.

In order to investigate the impact of the DNA extraction procedure on the microbial diversity DGGE analysis was carried out as described before (Wagner et al., 2011) with a gradient of 35–70% (from 7.0 M urea- and 40% formamide-stock solutions) for gels for archaeal, bacterial, and fungal diversity. As a marker the gene ruler 100 bp DNA ladder (Life Technologies) was used. Gels were stained with silver nitrate and data analysis was performed using GelCompar II (applied maths) with dendrogram type ‘ward’, similarity coefficient – band-based ‘dice’, position tolerance ‘1%’.

Additionally, qPCR was performed in order to evaluate the abundance of total archaea in DNA extracts. For this purpose the archaeal primers 787f/1059r were used as well as optimized running conditions as described in Reitschuler et al. (2013). Quantitative PCR was performed with SensiMix SYBR No-ROX kit (Bioline) on a Corbett Life Science (Qiagen) Rotor-Gene 6000 system. All PCR reactions and approaches were performed at least in duplicates, with “no template controls” (NTCs) and positive controls using 2 μl of template DNA in a final volume of 20 μl. As a standard the purified PCR product derived from *Methanosarcina acetivorans* pure culture DNA was used in known concentrations as described in Reitschuler et al. (2014). For quantification and efficiency calculations, diluted standards were used and the CT (cycle threshold) values were plotted against the log of given templates to obtain standard graphs as described in Bustin et al. (2009). The efficiency as well as melt curve analysis of the qPCR reactions were calculated by the Rotor-Gene software.

### 2.7 Statistical analysis

Statistical analysis was performed by using the Software package *Statistica 9.0* (StatSoft^®^) and *SigmaPlot 12.0* (Systat Software Inc.). Significant differences were ascertained by one-way or multifactorial ANOVA. A significance level of 0.05 was used to assess differences between treatments. *Fischers Least Significant Difference Test* was used to discriminate between single variants.

## 3 Results and discussion

### 3.1 Quantity and quality of DNA

**Fig. 2:**
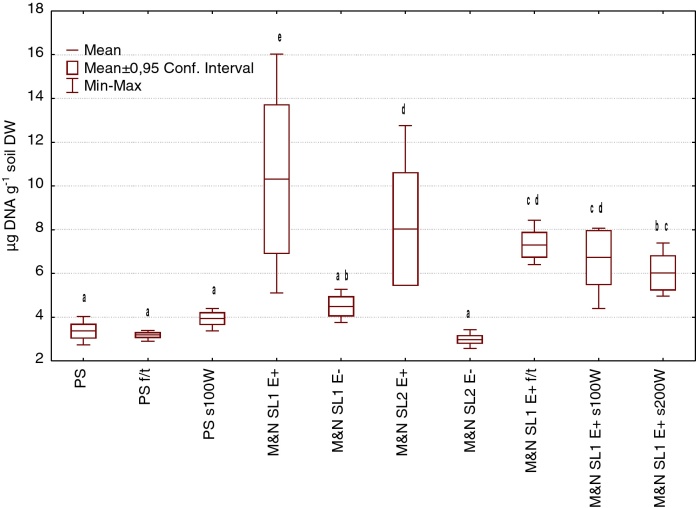
Total DNA yield using different DNA extraction protocols. PS: PowerSoil kit; M&N: SoilExtract II kit; SL1, SL2, E: M&N provided buffers and enhancer solution; f/t: freeze/thaw; s100 W: sonic treatment 100 W; s200 W: sonic treatment 200 W; *n* = 30.

The evaluation of total yielded DNA by the different DNA extraction protocols turned out to be difficult and tricky because of problems related to the comparison of different basal methods of DNA quantification (fluorometrically vs. spectrophotometrically). Although a close correlation of both methods was found (*R*^2^ = 0.87, data not shown) the variation of spectrophotometrical quantification in the range of <20 ng DNA μl^−1^ turned out to be unreliable. Therefore, all measurements were performed using both the methods. Data obtained fluorometrically were used for quantification of DNA as the fluorescent dye intercalates in double-stranded DNA specifically and thus provides very reliable results. The quality of the DNA extracts, however, was assessed using the spectrophotometrical data as the absorbance ratios indicate possible contaminations. Total DNA yields from the respective protocols are depicted in Fig. 2. This total yield accounts for the amount of DNA that could be extracted using the applied setup and conditions. However, it has to be kept in mind that a portion of DNA is lost during DNA purification steps throughout the DNA extraction procedure (Fornasier et al., 2014) and a high portion – Chroňáková et al. (2013) found 18–31% – of total DNA is derived from extracellular DNA. This portion of DNA is known to be quite stable (Agnelli et al., 2004).

Significant differences in the extraction efficiency were caused by the different extraction procedures and disintegration strategies (Fig. 2). The application of M&N kit using buffer SL1 and the highest manufacturer recommended enhancer concentration resulted in the highest DNA yield compared to all other tested protocols. Especially, the application of the enhancer solution as provided by the M&N kit had a highly significant effect on the total obtainable DNA yield but also increased the variation (Fig. 2) and the co-extraction of contaminants. A difference between PS and M&N kit extracted samples was also found. Soil samples extracted with M&N reached a significant higher total DNA yield than those with PS. Regardless of the used buffer system (TE, TGE, Crom) DNA extractions using phenol–chloroform led to believe in extremely high DNA yields (up to 0.4 mg DNA g^−1^ soil) when quantified spectrophotometrically, but PicoGreen measurements led to contradictory results. Humic substances and other contaminants interfere with spectrophotometric measurements due to their co-absorbance at similar wave-length of DNA (260 nm). The high values for DNA concentration from phenol–chloroform extractions could be attributed to this phenomenon. Thus, these data are not presented in Fig. 2. The presence of high amounts of those contaminants could be proven by the fact that the PCR amplification of extracted DNA was only possible after a dilution of at least 1:50 (see also below).

Disintegration strategies were applied for the most successful extraction protocols PS and M&N (using buffer SL1 and enhancer solution). Interestingly, their effect remained rather unremarkable and a significant increase in total DNA caused by their application could not be found. However, the statistical variation of samples extracted with M&N including enhancer solution was reduced by the application of the disintegration strategies.

The fragmentation of the extracted DNA was checked via gel electrophoresis and HPLC analysis since additionally applied disintegration strategies may lead to a higher degree of DNA fragmentation. A hypothesized strong effect in particular of the two applied sonication treatments (100 W and 200 W) could not be observed, although HPLC analysis pointed to a higher portion of fragments <10,000 bp (data not shown).

The quality of all DNA extracts was assessed by NanoDrop measurement. Although the interpretation of these results has to be carried out very carefully (Wilfinger et al., 1997), it can give hints on the “purity” of the extracted DNA. However, it is also dependent on the overall composition of the nucleic acid due to the different absorbance of nucleotides. Humic substance and protein co-extraction was assessed using the absorbance ratio A260/230, whereas the ratio A260/280 indicated co-extraction of proteins and high amounts of RNA. Generally ratios indicating good (acceptable) DNA quality – for A260/230 = 1.5–1.8 and A260/280 = 1.8–1.9 – could not be obtained for the first extraction, but a trend towards higher purity came up if no disintegration strategy was applied (data not shown). However, if subsequent extractions were performed (Section 3.1.1), the portion of co-extracted contaminants was reduced as reflected by ratios, which then indicated good DNA quality. In particular humic substance co-extraction (A260/280) seemed to decrease in the course of the 3 times-repeated DNA extractions.

From the tested DNA extraction protocols the kit extraction using M&N including buffer SL1 and enhancer solution without any additional disintegration strategy gave the highest total DNA yield as compared to the other tested protocols and variations. Therefore, subsequent experiments e.g., EMA/PMA treatment were performed using this DNA extraction protocol.

#### 3.1.1 Repeated DNA extraction

**Fig. 3:**
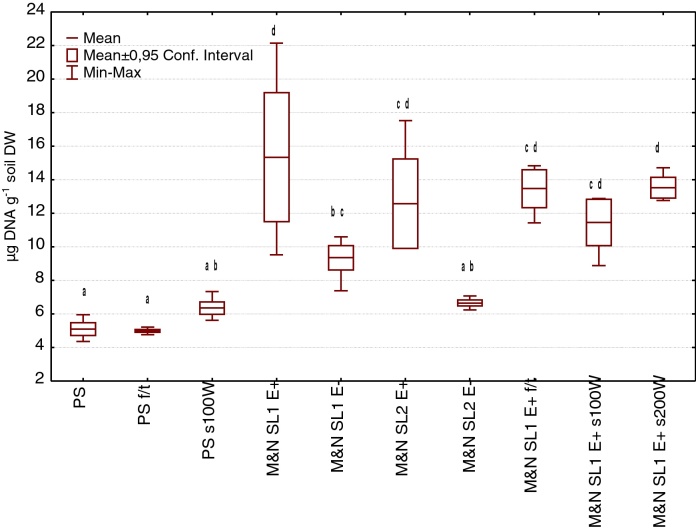
Total DNA yield using different DNA extraction protocols. Values derived from a 3 times repeated DNA extraction. PS: PowerSoil kit; M&N: SoilExtract II kit; SL1, SL2, E: M&N provided buffers and enhancer solution; f/t: freeze/thaw; s100 W: sonic treatment 100 W; s200 W: sonic treatment 200 W; *n* = 30.

In order to estimate the efficiency of all the tested protocols, DNA extractions were repeated 3 times and the amount of DNA was added up. By a 3 times repeated extraction the amount of extracted DNA could be increased (Fig. 3). Taking the total of the 3 extractions as a basis for the respective extraction protocol, the portion of DNA that could be extracted by the first extraction varied considerably. For the PS as well as the M&N extracted samples that included enhancer but no additional disintegration, a mean of more than 60% of DNA was obtained after the first extraction, whereas for samples extracted without enhancer the portion was reduced down to 45%. By the application of disintegration strategies using M&N kit the extraction efficiency was also reduced for the first extraction. Generally, the variation of the different extraction efficiencies in particular for the first extraction was high, illustrating the complex interactions taking place during a DNA extraction.

**Fig. 4:**
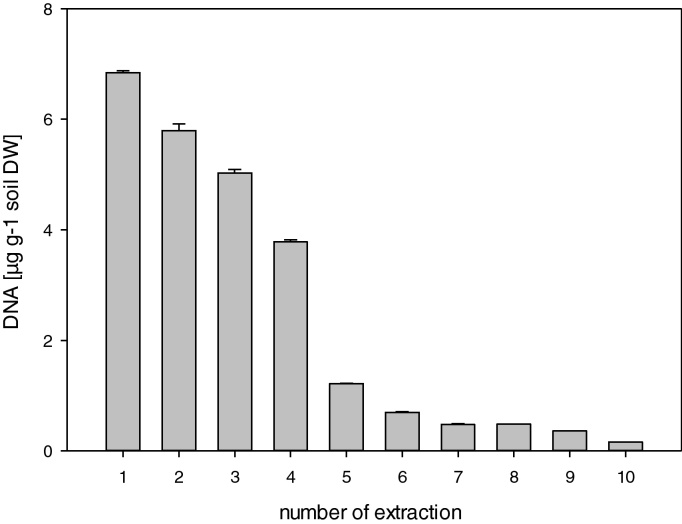
Amount of extractable DNA [μg g^−1^ soil DW] using SoilExtract II kit including buffer SL1 and enhancer during a 10 times extraction, *n* = 30.

In a separate experiment a 10 times repeated DNA extraction was performed using M&N extraction kit. Taking the total amount of DNA after the 10 times repeated extraction as a basis (100% total extractable DNA under the applied setup and conditions) after the 3rd and 5th extraction 71% and 91%, respectively, of the total extracted DNA was found. After 4 extractions the efficiency dropped distinctly and after 9 repeated extractions 99% of extractable DNA could be retrieved from the reference soil (Fig. 4) (see also below).

### 3.2 Microbial diversity

**Fig. 5:**
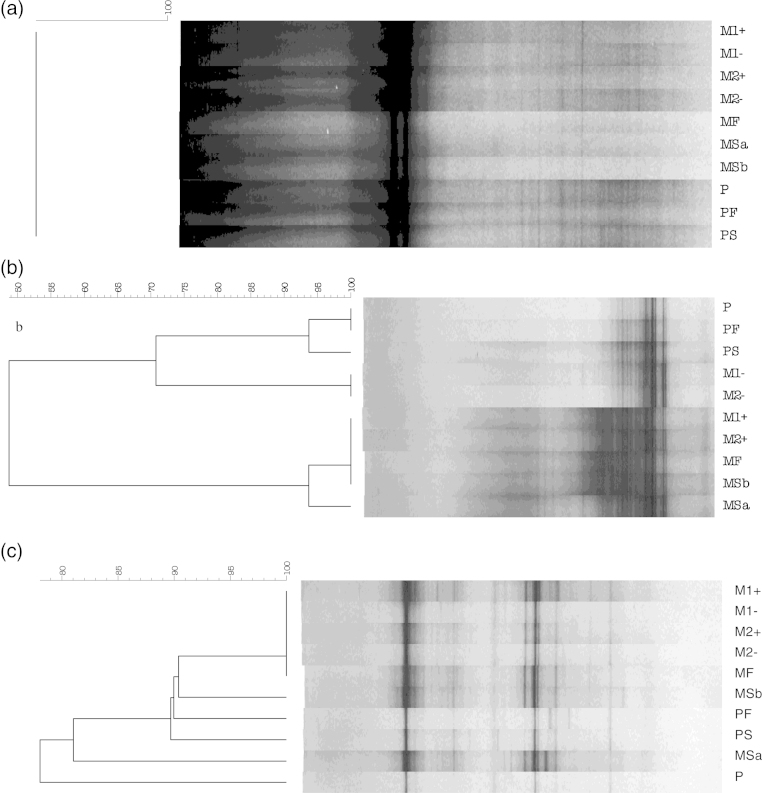
GelCompar analysis derived from DGGE of bacterial (a), archaeal (b), and fungal (c) diversity as obtained by different DNA extraction procedures. (P: power soil without treatment, PF: power soil freeze/thaw, PS: power soil sonication, M1: Macherey&Nagel SL1 (±enhancer solution), M2: Macherey&Nagel SL2 (±enhancer solution), MF: Macherey&Nagel freeze/thaw, MSa: Macherey&Nagel sonication 100 W, MSb: Macherey&Nagel sonication 200 W.

The DNA extraction method was impacting microbial fingerprints. A direct influence of the total amount of extracted DNA was expected, namely by a higher diversity in samples with higher DNA amounts. However, the influence was far less than expected indicating that the amount of total extracted DNA was primarily not determined by DNA originating from different organisms but rather from a higher extraction efficiency. On the contrary the different extraction protocols impacted the fingerprints for bacteria, archaea, and fungi in a different way (Fig. 5). As revealed by cluster analysis, DGGE profiles for bacteria were merely impacted by the extraction protocols and generally showed many but very weak bands. For archaea a clustering of samples extracted with M&N including enhancer solution was found (Fig. 5b, cluster A) being different from those extracted without enhancer or by PS kit (Fig. 5b, cluster B). On one hand the application of enhancer solution for M&N kit increased the number of DGGE bands (diversity) for archaea but also showed smearing bands on DGGE gels indicating the co-extraction of contaminants and therefore a biased PCR. For fungi, differences were not that distinct, although a clustering of M&N extracted samples was found, however, the application of enhancer did not alter these DGGE profiles significantly (Fig. 5c).

In order to prove the effect of DNA extraction protocols on the archaeal abundance, a qPCR approach was applied using archaeal primers. Results are depicted in Table 2 showing that PS and M&N without enhancer extracted samples resulted in a lower copy number than M&N extracted samples including the application of enhancer solution. Therefore, contrary to the diversity, the obtained abundance of the archaeal soil community was affected by the extraction method and in particular by the application of M&N enhancer solution, proving the above named hypothesis at least for archaea.

**Table 2:** Mean (±SD) of archaeal copy numbers [copies g^−1^ soil DW] for different soil DNA extraction protocols. PS: PowerSoil kit; M&N: SoilExtract II kit; SL1, SL2, E: M&N provided buffers and enhancer solution (E+, with; E−, without enhancer); f/t: freeze/thaw; s100 W: sonic treatment 100 W; s200 W: sonic treatment 200 W; *n* = 33.

Extraction procedure	Mean [copies g^−1^ soil]	±SD
PS	6.38 × 10^6^	1.13 × 10^3^
PS f/t	1.44 × 10^6^	2.97 × 10^3^
PS s100 W	1.12 × 10^6^	9.48 × 10^2^
M&N SL1E+	2.51 × 10^7^	1.84 × 10^4^
M&N SL1E−	4.37 × 10^6^	1.03 × 10^4^
M&N SL2E+	2.20 × 10^7^	5.37 × 10^4^
M&N SL2E−	2.01 × 10^6^	1.41 × 10^2^
M&N SL1E+	2.51 × 10^7^	1.84 × 10^4^
M&N SL1E+ f/t	3.25 × 10^7^	9.90 × 10^3^
M&N SL1E+ s100 W	3.07 × 10^7^	2.12 × 10^4^
M&N SL1E+ s200 W	1.70 × 10^7^	2.06 × 10^4^

#### 3.2.1 Repeated extraction

**Fig. 6:**
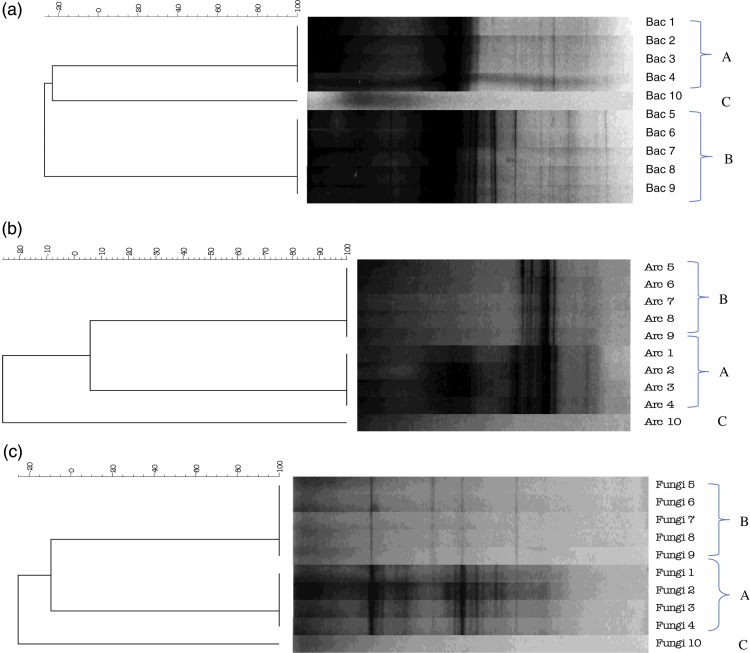
GelCompare analysis derived from DGGE of (a) bacterial (Bac), (b) archaeal (Arc), and (c) fungal (fungi) diversity as obtained by sequential DNA extraction procedures (10 fold extraction). Numbers indicate the number of the resprective extraction.

Cluster analysis of DGGE band profiles revealed significant differences in band patterns (number of bands reflecting the microbial diversity) for bacteria, archaea, and fungi. A clustering of the extractions 1–4 and 5–9, respectively, was found (Fig. 6). For the 10th extraction yielding 161 ng DNA g^−1^ soil (±5.8) no PCR product could be obtained. Interestingly, there was an opposite trend for bacteria on one hand and archaea and fungi on the other hand. As also observed when investigating the impact of the different extraction procedures, for sequential extractions 1–4 bacterial DGGE bands were weak, whereas this changed with the 5th extraction when additional bands appeared indicating that during the first 4 extractions only a part of the real bacterial diversity was captured (Fig. 6a). On the contrary, archaeal and fungal fingerprints showed the opposite trend (Fig. 6a and b). The diversity was high and not changing during the extractions 1–4, whereas it was reduced for extractions 5–9. For example, for the extractions 1–4 for archaea 27 bands were found while for extractions 5–9 only 14 bands could be detected on DGGE gels. Therefore, the drop in the extraction efficiency as observed for total DNA was also reflected by a distinct change in the detectable microbial diversity, but in an opposite manner for bacteria, archaea and fungi, respectively.

### 3.3 Unprotected DNA in soil

By the application of EMA and PMA during DNA extraction the portion of co-extracted unprotected DNA e.g., from dead organisms not maintaining cell wall integrity was attempted to be reduced or even avoided. Since DNA from cells overtaken by cell death can persist in soil for varying length of time maintaining sufficient molecular integrity (Chroňáková et al., 2013, England et al., 1997, England and Trevors, 2003, Herdina et al., 2004, Romanowski et al., 1993), the minimization of the co-extraction is a major target when extracting DNA in order not to overestimate the diversity (and depending on the downstream analysis, also the abundance) of the active population of a certain habitat. In the present study a significant effect of EMA treatment on the total DNA yield from reference soil was found. A similar trend was observed for PMA, although to a smaller extent. When EMA treatment was applied during DNA extraction using the optimized extraction procedure a reduction of 16.4% (0.74 μg g^−1^ soil) was achieved attributed to a decrease in the co-extraction of unprotected DNA. For PMA treatment the DNA yield was diminished of 14.9% (0.56 μg g^−1^ soil). Taken into account an average of 5 fg total DNA per microorganism (Rudd et al., 1990) this would represent a maximum reduction of approximately 7.4 × 10^8^ and 5.6 × 10^8^ microorganisms per gram soil for EMA and PMA treatment, respectively. However, not the complete portion of unprotected DNA in soil originates from microorganisms (Levy-Booth et al., 2007) and concentrations of extracellular DNA in the range of 0.03–200 μg g^−1^ soil are known (Pietramellara et al., 2009).

**Fig. 7:**
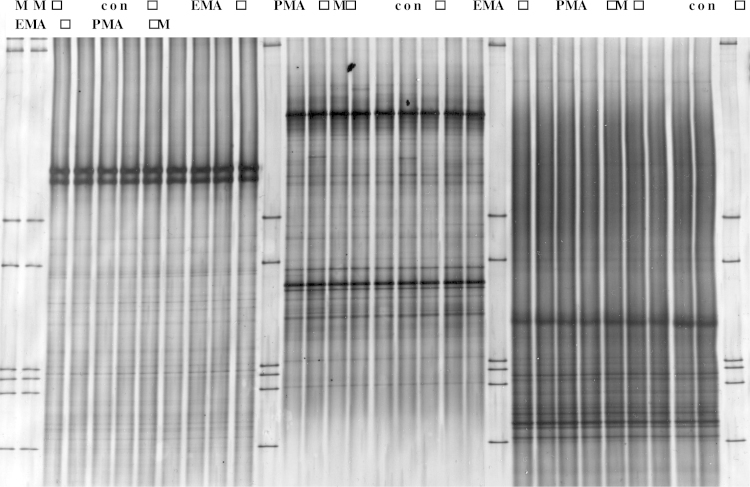
DGGE analysis of soil DNA extraction using additional EMA and PMA treatment, respectively. Depicted are 3 replicates of conventional DNA extraction (con), with additional EMA (EMA), and PMA treatment (PMA), respectively, for bacteria, fungi, and archaea (left to right), separated by marker lanes (M).

The impact of unprotected DNA on the total soil metagenome and therefore on subsequent analysis (e.g., microbial fingerprints) is discussed controversially (Nielsen et al., 2006, Niemeyer and Gessler, 2002) but a rather high impact was hypothesized at the beginning of this study. However, DGGE band-patterns of DNA extracts run through EMA/PMA treatment (Fig. 7) did not show significant differences as revealed by GelCompar analysis (please refer to Supplementary material). Solely, DGGE band-patterns for fungi showed slight alterations, however, this was rather indiscriminative. Thus, a hypothesized impact of unprotected DNA on the microbial community composition as derived from fingerprints could be refused. Generally, the reduction of DNA yield obtained after EMA/PMA treatment seems rather meager, since other studies (although in different matrices) showed a higher impact of EMA and PMA treatment on total DNA yield, respectively (Wagner et al., 2008). However, the interpretation of these data has to be carried out very carefully (Fittipaldi et al., 2011, Pacholewicz et al., 2013). Therefore, the impact of e.g., soil particles and high surface areas (Yu et al., 2013) as well as high amounts of dead cells (Fittipaldi et al., 2012) within the tested soil have to be evaluated in further studies in order to step forward to a better estimation of the real active population in different soil habitats using DNA extraction including EMA/PMA treatment as a basic technique.

## 4 Conclusion

A wide difference was found in the total extractable DNA from the investigated reference soil as derived from different extraction protocols. Concerning the DNA yield phenol–chloroform–isomyl alcohol extraction resulted in doubtfully high results and in a noteworthy co-extraction of contaminants making PCR from undiluted DNA extracts impossible. By comparison of two different extraction kits Macherey&Nagel SoilExtract II kit resulted in the highest DNA yields when buffer SL1 and the enhancer solution were applied. The enhancer solution not only significantly increased the DNA yield but also the amount of co-extracted contaminants, whereas additional disintegration strategies did not. Although a 3 times repeated DNA extraction increased the total amount of extracted DNA, microbial fingerprints were merely affected until the 5th extraction. After the fifth extraction a reduction of total DGGE band numbers was observed for archaea and fungi, whereas for bacteria the diversity increased. The application of EMA/PMA treatment, aiming on the selective removal of unprotected DNA in soil, resulted in a significant reduction of total extracted DNA, however, the hypothesized effect on microbial fingerprints failed to appear indicating the need for further investigations.

## References

[bib0005] Agnelli A., Ascher J., Corti G., Ceccherini M.T., Nannipieri P., Pietramellara G. (2004). Distribution of microbial communities in a forest soil profile investigated by microbial biomass, soil respiration and DGGE of total and extracellular DNA. Soil Biol. Biochem..

[bib0010] Ascher J., Ceccherini M.T., Pantani O.-L., Agnelli A., Borgogni F., Guerri G., Nannipieri P., Pietramellara G. (2009). Sequential extraction and genetic fingerprinting of a forest soil metagenome. Appl. Soil Ecol..

[bib0015] Belasco J., Belasco J., Brawerman G. (1993). Control of Messenger RNA Stability.

[bib0020] Bustin S., Benes V., Garson J., Hellemans J., Huggett J., Kubista M., Mueller R., Nolan T., Pfaffl M., Shipley G., Vandesompele J., Wittwer C. (2009). The MIQE guidelines: minimum information for publication of quantitative real-time PCR experiments. Clin. Chem..

[bib0025] Ceccherini M.T., Ascher J., Agnelli A., Borgogni F., Pantani O.-L., Pietramellara G. (2009). Experimental discrimination and molecular characterization of the extracellular soil DNA fraction. Antonie van Leeuwenhoek.

[bib0030] Chroňáková A., Ascher J., Jirout J., Ceccherini M.T., Elhottová D., Pietramellara G., Šimek M. (2013). Evaluation of cattle impact on soil archaea, bacteria, and fungi by comparative fingerprinting of total and extracellular DNA. Biol. Fertil. Soils.

[bib0035] Elizaquivel P., Aznar R., Sanchez G. (2014). Recent developments in the use of viability dyes and quantitative PCR in the food microbiology field. J. Appl. Microbiol..

[bib0040] England L.S., Trevors J.T. (2003). The microbial DNA cycle in soil. Riv. Biol..

[bib0045] England L.S., Lee H., Trevors J.T. (1997). Persistence of *Pseudomonas aureofaciens* strains and DNA in soil. Soil Biol. Biochem..

[bib0050] Fittipaldi M., Codony F., Adrados B., Camper A.K., Morato J. (2011). Viable real-time PCR in environmental samples: can all database be interpreted directly?. Microb. Ecol..

[bib0055] Fittipaldi M., Nocker A., Codony F. (2012). Progress in understanding preferencial detection of live cells using viability dyes in combination with DNA amplification. J. Microbiol. Methods.

[bib0060] Fornasier F., Ascher J., Ceccherini M.T., Tomat E., Pietramellara G. (2014). A simplified rapid, low-cost and versatile DNA-based assessment of soil microbial biomass. Ecol. Indic..

[bib0065] Hazen T.C., Rocha A.M., Techtmann S.M. (2013). Advances in monitoring environmental microbes. Curr. Opin. Biotechnol..

[bib0070] Herdina M., Neate S., Jabaji-Hare S., Ophel-Keller K. (2004). Persistence of DNA of *Gaeumannomyces graminis* var. tritici in soil as measured by a DNA-based assay. FEMS Microbiol. Ecol..

[bib0075] Juen A., Traugott M. (2005). Detecting predation and scavenging by DNA gut-content analysis: a case study using a soil insect predator–prey system. Oecologia.

[bib0080] Krsek M., Wellington E.M.H. (1999). Comparison of different methods for the isolation and purification of total community DNA from soil. J. Microbiol. Methods.

[bib0085] Lee J.-L., Levin R.E. (2007). Quantification of total viable bacteria on fish fillets by using ethidium bromide monoazide real-time polymerase chain reaction. Int. J. Food Microbiol..

[bib0090] Levy-Booth D.J., Campell R.G., Gulden R.H., Hart M.M., Powell J.R., Klironomos J.N., Pauls K.P., Swanton C.J., Trevors J.T., Dunfield K.E. (2007). Cycling of extracellular DNA in the soil environment. Soil Biol. Biochem..

[bib0095] Maciel B.M., Santos A.C.F., Dias J.C.T., Vidal R.O., Dias R.J.C., Gross E., Cascardo J.C.M., Rezende R.P. (2009). Simple DNA extraction protocol for a 16S rDNA study of bacterial diversity in trophic landfarm soil used for bioremediation of oil waste. Genet. Mol. Res..

[bib0100] Nielsen K.M., Calamai L., Pietramellara G., Nannipieri P., Smalla K. (2006). Nucleic Acids and Proteins in Soil (Soil Biology).

[bib0105] Niemeyer J., Gessler F. (2002). Determination of free DNA in soils. J. Plant Nutr. Soil Sci..

[bib0110] Nocker A., Camper A.K. (2006). Selective removal of DNA from dead cells of mixed bacterial communities by use of ethidium monoacide. Appl. Environ. Microbiol..

[bib0115] Nocker A., Camper A.K. (2009). Novel approaches toward preferential detection of viable cells during nucleic acid amplification techniques. FEMS Microbiol. Lett..

[bib0120] Nocker A., Cheung C.Y., Camper A.K. (2006). Comparison of propidium monoazide with ethidium monoazide for differentiation of live vs. dead bacteria by selective removal of DNA from dead cells. J. Microbiol. Methods.

[bib0125] Ogram A.V., Mathot M.L., Harsh J.B., Boyle J., Pettigrew J.C.A. (1994). Effects of DNA polymer length on its adsorption to soils. Appl. Environ. Microbiol..

[bib0130] Pacholewicz E., Swart A., Lipman L.J.A., Wagenaar J.A., Havelaar A.H., Duim B. (2013). Propidium monoazide does not fully inhibit the detection of dead *Campylobacter* on broiler chicken carcasses by qPCR. J. Microbiol. Methods.

[bib0135] Pietramellara G., Ascher J., Borgogni F., Ceccherini M.T., Guerri G., Nannipieri P. (2009). Extracellular DNA in soil and sediment: fate and ecological relevance. Biol. Fertil. Soils.

[bib0140] Reitschuler C., Lins P., Illmer P. (2013). Primer evaluation and adaption for cost-efficient SYBR green-based qPCR and its applicability for specific quantification of methanogens. World J. Microbiol. Biotechnol..

[bib0145] Reitschuler C., Lins P., Wagner A.O., Illmer P. (2014). Cultivation of moonmilk-born non-extremophilic thaum- and euryarchaeota in mixed culture. Anaerobe.

[bib0150] Romanowski G., Lorenz M.G., Wackernagel W. (1993). Use of polymerase chain reaction and electroporation of *Escherichia coli* to monitor the persistence of extracellular plasmid DNA introduced into natural soils. Appl. Environ. Microbiol..

[bib0155] Rudd K.E., Miller W., Ostell J., Benson D.A. (1990). Alignment of *Escherichia coli* K12 DNA sequences to a genomic restriction map. Nucleic Acids Res..

[bib0160] Rudi K., Moen B., Dromtorp M., Holck A.L. (2005). Use of ethidium monoazide and PCR in combination for quantification of viable and dead cells in complex samples. Appl. Environ. Microbiol..

[bib0165] Shi H., Xu W., Luo Y., Chen L., Liang Z., Zhou X., Huang K. (2011). The effect of various environmental factors on the ethidium monazite and quantitative PCR method to detect viable bacteria. J. Appl. Microbiol..

[bib0170] Smith C.J., Osborn A.M. (2009). Advantages and limitations of quantitative PCR (Q-PCR)-based approaches in microbial ecology. FEMS Microbiol. Ecol..

[bib0175] Taberlet P., Coissac E., Hajibabaei M., Riesberg L.H. (2012). Environmental DNA. Mol. Ecol..

[bib0180] Trevors J.T. (2012). Can dead bacterial cells be defined and are genes expressed after cell death?. J. Microbiol. Methods.

[bib0185] van Frankenhuyzen J.K., Trevors J.T., Flemming C.A., Lee H., Habash M.B. (2013). Optimization validation, and application of a real-time PCR protocol for quantification of viable bacterial cells in municipal sewage sludge and biosolids using reporter genes and *Escherichia coli*. J. Ind. Microbiol. Biotechnol..

[bib0190] Vainio E.J., Hantula J. (2000). Direct analysis of wood-inhabiting fungi using denaturing gradient gel electrophoresis of amplified ribosomal DNA. Mycol. Res..

[bib0195] Wagner A.O., Malin C., Knapp B.A., Illmer P. (2008). Removal of free extracellular DNA in environmental samples by ethidium monoazide (EMA) and propidium monoazide (PMA). Appl. Environ. Microbiol..

[bib0200] Wagner A.O., Malin C., Illmer P. (2009). Application of denaturing high-performance liquid chromatography in microbial ecology: fermentor sludge, compost, and soil community profiling. Appl. Environ. Microbiol..

[bib0205] Wagner A.O., Malin C., Lins P., Illmer P. (2011). Effects of various fatty acid amendments on a microbial digester community in batch culture. Waste Manage..

[bib0210] Wilfinger W.W., Mackey K., Chomczynski P. (1997). Effect of pH and ionic strength on the spectrophotometric assessment of nucleic acid purity. BioTechniques.

[bib0215] Yu H.Q., Mu Y., Fang H.H.P. (2004). Thermodynamic analysis of product formation in mesophilic acidogenesis of lactose. Biotechnol. Bioeng..

[bib0220] Yu W.H., Li N., Tong D.S., Zhou C.H., Lin C.X., Xu C.Y. (2013). Adsorption of proteins and nucleic acids on clay minerals and their interactions: a review. Appl. Clay Sci..

