# Supplementary material for: Effect of DNA extraction procedure, repeated extraction and ethidium monoazide (EMA)/propidium monoazide (PMA) treatment on overall DNA yield and impact on microbial fingerprints for bacteria, fungi and archaea in a reference soil
Source: Appl Soil Ecol. 2015 Sep;93:56–64. doi: 10.1016/j.apsoil.2015.04.005 (PMC4461152; doi:10.1016/j.apsoil.2015.04.005)
Supplement: Supplementary file 1 [file mmc1.docx]

Supplementary: GelCompare analysis from DGGE analysis of soil DNA extraction using additional EMA and PMA treatment, respectively. Depicted are 3 replicates (1, 2, 3) of conventional DNA extraction, with additional EMA and PMA treatment for Bacteria (Bac), Fungi (Fun), and Archaea (Arc).
